# National Adolescent Treatment Trial for Obesity in Kuwait (NATTO): project design and results of a randomised controlled trial of a good practice approach to treatment of adolescent obesity in Kuwait

**DOI:** 10.1186/1745-6215-15-234

**Published:** 2014-06-19

**Authors:** Shurooq A Boodai, John H McColl, John J Reilly

**Affiliations:** 1 University of Glasgow School of Medicine, Level 3 New Lister Building, GRI, 10 Alexander Parade, Glasgow, Scotland; 2 University of Glasgow School of Mathematics and Statistics, 15 University Gardens, G12 8QQ Glasgow, Scotland; 3 University of Strathclyde Physical Activity for Health Group, School of Psychological Sciences & Health, Graham Hills Building (Room 531)50 George Street, G1 1QE Glasgow, Scotland

**Keywords:** obesity, overweight, adolescents, treatment, BMI, randomised controlled trial

## Abstract

**Background:**

Few randomised controlled trials (RCTs) of interventions for the treatment of adolescent obesity have taken place outside the western world. This RCT tested whether a simple ‘good practice’ intervention for the treatment of adolescent obesity would have a greater impact on weight status and other outcomes than a referral to primary care (control) in adolescents in Kuwait City.

**Methods:**

We report on an assessor-blinded RCT of a treatment intervention in 82 obese 10- to 14-year-olds (mean age 12.4, SD 1.2 years), randomised to a good practice treatment or primary care control group over 6 months. The good practice intervention was intended as relatively low intensity (6 hours contact over 24 weeks, group-based), aiming to change sedentary behaviour, physical activity, and diet. The primary outcome was a change in body mass index (BMI) Z score; other outcomes were changes in waist circumference and blood pressure.

**Results:**

The retention of subjects to follow up was acceptable (n = 31 from the intervention group, and n = 32 from the control group), but engagement with both the intervention and control treatment was poor. Treatment had no significant effect on BMI Z score relative to control, and no other significant benefits to intervention were observed.

**Conclusions:**

The trial was feasible, but highlights the need to engage obese adolescents and their families in the interventions being trialled. The trial should inform the development of future adolescent obesity treatment trials in the Gulf States with the incorporation of qualitative assessment in future intervention trials.

**Trial registration:**

RCT Registered as National Adolescent Treatment Trial for Obesity in Kuwait (NATTO): http://www.controlled-trials.com/ISRCTN37457227, 1 December 2009.

## Background

Prevalence of child and adolescent obesity has increased dramatically in recent years in Kuwait, [1,2], as in much of the rest of the world. While prevention of obesity is paramount, most preventive interventions have had only a modest impact, and there is a need to offer weight management interventions for adolescents who are already obese [3,4], particularly given the large number of serious short-term and long-term co-morbidities of adolescent obesity [5,6].

Despite the importance of treatment interventions for adolescent obesity, recent systematic reviews have found almost no evidence on treatment interventions outside the western world [3]. Specifically, the recent Cochrane review [3] found 29 eligible trials of adolescent obesity treatment, and none of these were from the Arab world (16 from North America, 7 from Europe and Australia; 3 from Asia; and 2 from Israel). The primary aim of the present study was therefore to test the hypothesis that a ‘good practice’ intervention for the treatment of adolescent obesity in Kuwait would have a greater effect on primary and secondary outcomes than allocation to a control group. The secondary aims were to test the feasibility of conducting such a trial in Kuwait and to test the feasibility of using a good practice intervention and referral to primary care as a control condition, with a view to developing improved obesity treatment RCTs in Kuwait and the other Gulf States.

## Methods

### Participants

The study was conducted at Al Faiha polyclinic (primary care clinic) during 2009. For entry into the study, adolescents, ages 10 to 14 years had to be obese (body mass index (BMI) above the 95^th^ percentile [7]), have at least one parent who expressed a willingness to attend the intervention described below if allocated to it, and have no serious underlying medical condition that might be either a cause or consequence of their obesity. Adolescents were recruited from their schools after BMI and health screening was conducted by one of the researchers (SAB). The schools and residences of all participants were located in the capital, and both the venue for the intervention (Al Faiha polyclinic) and the venue for the control primary care clinic were also in the capital to ensure that distance was not an issue hindering attendance. Ethical approval was obtained from the Ethics Committee for Medical Research, Ministry of Health of Kuwait (ref MPH/112), and written informed consent was obtained from both parents and adolescents.

### Randomisation and allocation concealment

Participating adolescents attended a research clinic where all baseline measures (see below) were taken, then assigned a unique study code prior to random allocation into the treatment or control group. To ensure concealment of allocation, codes were sent electronically to a statistician (JHM) who produced a computer generated randomisation list that allocated participants to intervention or control group, with participants balanced for gender in blocks of 10. The statistician informed the researcher responsible for delivering the intervention (SAB) of the allocation, and families were invited to intervention or control groups as appropriate.

### Intervention

In brief, the intervention was intended as a relatively low intensity (6 sessions, 1 hour contact time per session, delivered as a group session) programme, which might be readily generalisable if evidence of feasibility and efficacy was obtained from the present study. The intervention was delivered to the adolescents and their parents in group-discussion fashion over a 24-week period by a physician with specialist training in Nutrition (SAB) and the study dietician. The programme was adapted from the Scottish Childhood Obesity Treatment Trial (SCOTT), which tested a ‘good practice’ treatment intervention in Scotland [8,9]. Parents were provided with treatment materials that were adapted from those used in SCOTT [9]. The intervention is described here as a ‘good practice’ intervention on two grounds. First, because it focused on changing the behaviours recommended as the key targets in recent evidence-based management guidelines [10-12] for the treatment of adolescent obesity (reduction in sedentary behaviour, particularly screen-media use; diet, using a modified version of the ‘traffic light diet’ system [8]; and promotion of physical activity). Second, the intervention incorporated theoretically based behaviour change techniques to all three of the targeted behaviours [8]: exploration of the pros and cons of changes in diet, physical activity, and sedentary behaviour; exploration of motivation to change diet, physical activity, and sedentary behaviour; self-monitoring of sedentary behaviour (recording of screen time in diaries), diet, and physical activity (recording of walking, sport, and physically active play in a diary); identifying the main barriers to behaviour change and problem solving in relation to these barriers; goal setting in relation to diet, physical activity, and sedentary behaviour; and relapse prevention.

The intervention group was further divided into boys (n = 21, each attending with at least one parent) and girls (n = 20, each attending with at least one parent) groups in accordance with cultural norms of the Kuwaiti population, and their sessions were delivered on two consecutive days. Any adolescent who attended the intervention session alone was welcomed, although ideally at least one parent should have been present.

### Control group

Primary care-based treatment of child and adolescent obesity in Kuwait is somewhat limited, as in many other countries, but it was felt ethically and scientifically appropriate to use referral to primary care as a control condition in the present study. Adolescents, and their parents, who were allocated randomly to the control group were therefore informed that they were obese and advised to attend primary care.

### Outcome measures and blinding

BMI Z scores were calculated based on US CDC 2000 reference data [7] using the software available at http://stokes.chop.edu/web/zscore/index.php. Outcome measures were made at baseline and again at 6 months (26 weeks) after the start of the intervention by the same trained research assistants who were blinded to group allocation and were not involved in delivery of the treatment intervention. Blood pressure was measured when the participant was sitting quietly in the upright position, with the correct cuff size applied to the right arm. The reading was repeated three times and the average of the three readings was taken.

The primary study outcome measure was change in BMI Z score. Weight was measured to 0.1 kg in light indoor clothing with children not wearing shoes, and height was measured to 0.1 cm with a portable stadiometer (Leicester Height Measure, SECA, London, UK) and adolescents not wearing shoes. Secondary outcomes were waist circumference and blood pressure.

### Sample size, power, and statistical analysis

No local data were available upon which to base a power calculation. The present study was therefore powered using BMI data from the Scottish SCOTT RCT [8], which was used to develop the treatment intervention. With a between-group difference in the change in BMI Z-score of -0.25 at 6 months (which is a small change in BMI Z score, as discussed below) and a SD of change in BMI Z score of 0.21, giving a delta of 1.15, a sample size of around 30 adolescents per arm at 6 months would give 90% power at the 0.05 significance level. Dropout from the trial could not be predicted, but it was hoped that entering around 90 adolescents would make sufficient allowance for attrition during the 6-month study to leave around 30 participants per arm at the end of the trial.

Outcomes were analysed in two ways. First, changes in outcome variables within each group (intervention and control) between baseline and 6-month follow-up are presented. The issue of whether changes in outcome variables differed significantly between groups (intervention versus control) was examined using independent sample t-tests. The analysis was intention-to-treat, where we used data from all adolescents for whom data were available on the basis of the group to which they were allocated, regardless of their adherence to the protocol (attendance).

### Feasibility of the trial: treatment intervention and control conditions

Since the present study was the first of its kind in Kuwait, it was not considered as a ‘definitive trial’ [13] but rather as the initiation of a process which should lead to a more definitive trial subsequently [13]. Feasibility of the trial and feasibility of the trial interventions were measured by summarising the extent of sample attrition (dropout) over the 6-month period, the extent of missing data, and recording attendance at intervention and control treatments.

## Results

### Flow of participants through the trial, flow through the interventions, and participant characteristics

**Figure 1:**
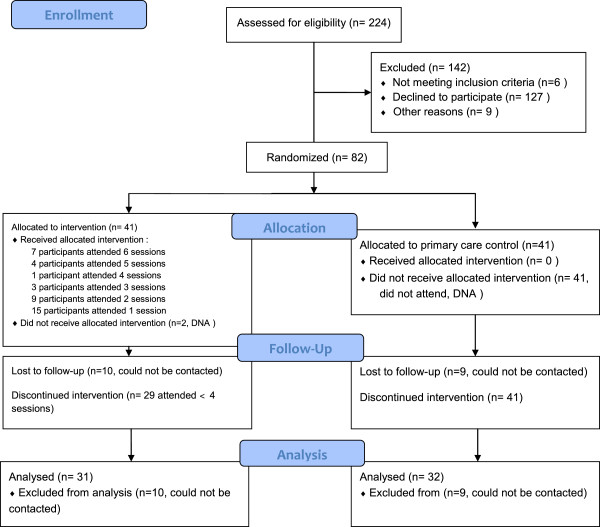
CONSORT 2010 flow diagram.

Figure 1 describes the flow of participants through the trial and through the intervention. Of the 82 participants entered at baseline, 63 (77%) attended for outcome measures at the 6-month follow-up. There were no significant differences at baseline between intervention and control groups for adolescent age, anthropometric measures and weight status (Table 1).

**Table 1:** Characteristics of participating adolescents at baseline

**Characteristic**	**Full sample**	**Treatment group**	**Control group**
	**n = 82**	**n = 41**	**n = 41**
	**Mean (SD)**	**Mean (SD)**	**Mean (SD)**
Age (years)	12.4 (1.2)	12.4 (1.2)	12.4 (1.2)
Male/female	42/40	21/20	21/20
Body mass index Z score	2.2 (0.3)	2.2 (0.3)	2.2 (0.3)
Systolic blood pressure (mmHg)	122.2 (10.4)	122.0 (12.3)	122.0 (8.2)
Diastolic blood pressure	77.5 (7.6)	76.0 (8.5)	79.1 (6.4)
Waist circumference (cm)	93.5 (12.1)	93.0 (12.7)	94.0 (11.6)

Attendance at the intervention group and control group sessions was low and is shown in Figure 1.

### Changes in primary and secondary outcomes within and between groups

Table 2 provides data on change in BMI Z scores, blood pressures and waist circumference. There were no statistically significant differences within the two groups over the 6 months for any of the anthropometric measures. There were also no significant differences between the groups for the 6-month changes in anthropometry (Table 2). Only 7 of the 31 adolescents in the intervention group at the end of the study maintained or lost weight over the 6 months, while 5/32 in the control group maintained or lost weight over the 6 months.

**Table 2:** Six-month changes in outcome measures within and between-groups (n = 31 treatment group versus 32 controls)

**Outcome**	**Intervention group within-group change mean (SD)**	**Control group within-group change mean (SD)**	**Between-group difference, mean (95% CI), *P *** **value**
Body mass index Z score	0.0 (0.1)	0.0 (0.2)	0.0 (-0.1; 0.1), 0.6
Systolic blood pressure (mmHg)	0.4 (6.7)	0.6 (4.8)	0.3 (-2.7; 3.2), 0.9
Diastolic blood pressure (mmHg)	2.9 (6.2)	1.1 (5.8)	-1.8 (-4.8, 1.3), 0.2
Waist circumference (cm)	4.9 (5.8)	3.5 (5.7)	-1.4 (-4.3; 1.5), 0.3

## Discussion

### Main findings, study implications, and comparisons with other evidence

While it seems that no previous adolescent obesity treatment RCTs have been published from Kuwait or the other Gulf States, the present study suggests that conducting randomised controlled trials of adolescent obesity treatment interventions in Kuwait is feasible. Retention in the trial was acceptable and not strikingly dissimilar to that reported in other adolescent obesity treatment trials that took place over a similar timescale [3]. Luttikhuis and colleagues in the recent Cochrane review described attrition in the eligible adolescent obesity treatment trials (of at least 6 months duration) as ranging from 0 to 43% by the end of the intervention [3]. An expansion of the evidence base on interventions to treat adolescent obesity is required because most obese adolescents now live outside western countries [14]. However, the recent Cochrane review [3] found no eligible RCT from the Arab world.

The present study also suggests that adherence to obesity interventions in obese adolescents and their families in Kuwait might be very poor. While retention in the trial was acceptable, engagement with the interventions offered was limited: only 12 participants (29% of the sample) attended ≥4 sessions, and the control group families did not attend any sessions at primary care. The reasons for poor adherence to both the study intervention and control treatments are probably complex and a detailed discussion of them is beyond the scope of the present study. However, a number of the adolescents and their families expressed a low degree of concern about obesity on being given the diagnosis and at the treatment sessions which they attended, and the poor attendance is consistent with this view. The fact that attendance was negligible even in the control group implies a low degree of concern about obesity rather than any specific non-engagement with the intervention arm of the trial. In a recent study of adolescents in Kuwait city [15] we reported that health-related quality of life was not impaired in obese adolescents relative to their healthy weight peers; this is an unusual finding [16,17], and it seemed to reflect a cultural difference between western and Kuwaiti societies, with a reduced concern over obesity in Kuwait [15]. Of note, we analysed blood samples from 80 out of the 82 participants in NATTO for cardiometabolic risk factors at baseline; however, results of the analyses were only available after the end of the trial. Had these results been available, they may have had an impact on the motivation to attend of both the intervention and the control groups. Several studies have shown multiple cardiometabolic risk factors in obese adolescents both in Kuwait [18] and internationally [19,20].

Exploring the reasons for non-attendance and non-adherence to treatment and investigating the treatment preferences of obese adolescents and their families would be important for future adolescent obesity treatment research in Kuwait and the other Gulf States. Indeed, the UK Medical Research Council Framework on the Development and Evaluation of Complex Interventions [13] recommends an approach in which interventions are developed in conjunction with study participants, and qualitative studies are used to understand treatment preferences.

The degree of change in body weight status that might be desirable in an adolescent obesity treatment intervention is uncertain [10-12], but improvements in cardiometabolic risk factors probably require much greater changes than were observed in the present study, [21,22]. Weight maintenance or modest weight loss is usually recommended for adolescent obesity treatment interventions [10-12], but in the present study, only a minority of participants maintained or lost weight. Since adherence to treatment ranged from limited to absent in the present study, the actual ‘dose’ of obesity treatment was probably also very low, amounting to little more than the confirmation to the adolescents and their families that they were obese with some evidence-based advice. The present study is therefore consistent with some others (for example, [23]) in suggesting that diagnosis of obesity plus good advice alone is likely to have null or minimal effects on energy balance of obese adolescents.

### Study strengths and weaknesses

The principal strengths of the present study were the high level evidence obtained, with adherence to the CONSORT statement on conduct and reporting of RCTs [24]; the fact that the trial was powered adequately, in contrast to a number of previous trials in this area [3]; development and testing of a potentially generalisable intervention; and completing a challenging adolescent obesity treatment RCT [25] in the novel setting of a Gulf State.

The present study also had a number of weaknesses. Longer term obesity treatment trials are desirable, and a 6-month follow-up is considered the minimum desirable in the most recent Cochrane review of paediatric obesity treatment RCTs [3]. An assessment of parent and adolescent perspectives on the treatment programme would have been desirable in order to both understand the current intervention better and to inform future treatment interventions [13,26,27]. Future intervention trials in Kuwait might also find it useful to focus treatment at other population subgroups (for example, younger or older participants, or to tailor treatment to boys or to girls), but this was not possible in the present study due to resource limitations.

## Conclusions

The present study suggests that trials of obesity treatment interventions in adolescents in Kuwait are feasible, but that the success of future trials will depend on addressing the problem of low adherence to treatment.

## Abbreviations

BMI: body mass index; BMI Z score: body mass index Z score; CONSORT: consolidated standards of reporting trials; RCT(s): randomised controlled trial(s); SD: standard deviation.

## Competing interests

The authors declare that they have not competing interests.

## Authors’ contributions

SAB: Conception and design, data collection and analysis, manuscript writing and final approval. JHM: Design, analysis, critical revision and final approval of the manuscript. JJR: Conception and design, manuscript writing and critical revision and final approval of the manuscript. All authors read and approved the final manuscript.
